# Limited Benefit from the Addition of Immunotherapy to Chemotherapy in TKI-Refractory EGFR-Mutant Lung Adenocarcinoma

**DOI:** 10.3390/cancers14143473

**Published:** 2022-07-17

**Authors:** Lingzhi Hong, Whitney E. Lewis, Monique Nilsson, Sonia Patel, Susan Varghese, Melvin J. Rivera, Robyn R. Du, Pingjun Chen, Haley N. Kemp, Waree Rinsurongkawong, Simon Heeke, Amy R. Spelman, Yasir Y. Elamin, Marcelo V. Negrao, Boris Sepesi, Don L. Gibbons, J. Jack Lee, Jia Wu, Natalie I. Vokes, John V. Heymach, Jianjun Zhang, Xiuning Le

**Affiliations:** 1 Department of Thoracic/Head and Neck Medical Oncology, The University of Texas MD Anderson Cancer Center, Houston, TX 77030, USA; lhong@mdanderson.org (L.H.); welewis@mdanderson.org (W.E.L.); mnilsson@mdanderson.org (M.N.); spatel24@mdanderson.org (S.P.); svarghese@mdanderson.org (S.V.); mjrivera@mdanderson.org (M.J.R.); rrdu@mdanderson.org (R.R.D.); hnkemp@mdanderson.org (H.N.K.); wcarter@mdanderson.org (W.R.); sheeke@mdanderson.org (S.H.); aspelman@mdanderson.org (A.R.S.); yyelamin@mdanderson.org (Y.Y.E.); mvnegrao@mdanderson.org (M.V.N.); dlgibbon@mdanderson.org (D.L.G.); jwu11@mdanderson.org (J.W.); nvokes@mdanderson.org (N.I.V.); jheymach@mdanderson.org (J.V.H.); jzhang20@mdanderson.org (J.Z.); 2 Department of Imaging Physics, The University of Texas MD Anderson Cancer Center, Houston, TX 77030, USA; pchen6@mdanderson.org; 3 Department of Thoracic and Cardiovascular Surgery, The University of Texas MD Anderson Cancer Center, Houston, TX 77030, USA; bsepesi@mdanderson.org; 4 Department of Biostatistics, The University of Texas MD Anderson Cancer Center, Houston, TX 77030, USA; jjlee@mdanderson.org; 5 Department of Genomic Medicine, The University of Texas MD Anderson Cancer Center, Houston, TX 77030, USA

**Keywords:** lung adenocarcinoma, EGFR, tyrosine kinase inhibitors, immunotherapy, chemotherapy

## Abstract

**Simple Summary:**

Although it is known that anti-PD1/L1 monotherapy does not render significant benefit in patients with EGFR-mutant lung adenocarcinoma, whether the addition of anti-PD1/L1 therapy to chemotherapy can enhance chemotherapy efficacy for TKI-refractory EGFR-mutant lung adenocarcinoma patients is not clear. To address this question, we retrospectively analyzed a cohort of 178 EGFR-mutant lung adenocarcinoma patients who had progressed on EGFR TKIs and received subsequent non-TKI systemic therapy to determine whether the addition of immunotherapy to chemotherapy truly improves clinical outcomes. We found that the addition of anti-PD1 immunotherapy did not add benefit to the platinum-based chemotherapy at the time of TKI progression for EGFR-mutant LUAD. Although underpowered, the anti-VEGF therapy demonstrated a trend towards adding benefit. As ongoing clinical trials with newer agents or combinations demonstrate preliminary efficacy in TKI-resistant EGFR-mutant LUAD patients, the ideal choice for post-TKI treatment is still being evaluated.

**Abstract:**

Background: The benefit of chemotherapy combined with immunotherapy in EGFR-mutant lung adenocarcinoma (LUAD) patients whose tumor developed resistance to EGFR tyrosine kinase inhibitors (TKIs) is not thoroughly investigated. The goal of this retrospective cohort study is to assess the clinical efficiency of immunotherapy alone or in combination with chemotherapy in a real-world setting. Methods: This retrospective cohort study enrolled LUAD patients with EGFR sensitive mutations whose tumor had acquired resistance to EGFR TKIs and received systemic treatment with chemotherapy (chemo; *n* = 84), chemotherapy combined with immunotherapy (chemoIO; *n* = 30), chemotherapy plus bevacizumab with or without IO (withBev; *n* = 42), and IO monotherapy (IO-mono; *n* = 22). Clinical progression-free survival (PFS) and overall survival (OS) were evaluated. Associations of clinical characteristics with outcomes were assessed using univariable and multi-covariate Cox Proportional Hazards regression models. Results: A total of 178 patients (median age = 63.3; 57.9% females) with a median follow-up time of 42.0 (Interquartile range: 22.9–67.8) months were enrolled. There was no significant difference in PFS between chemoIO vs. chemo groups (5.3 vs. 4.8 months, *p* = 0.8). Compared to the chemo group, patients who received withBev therapy trended towards better PFS (6.1 months vs. 4.8; *p* = 0.3; HR 0.79; 95% CI: 0.52–1.20), while patients treated with IO-mono had inferior PFS (2.2 months; *p* = 0.001; HR 2.22; 95% CI: 1.37–3.59). Furthermore, PD-L1 level was not associated with PFS benefit in the chemoIO group. Patients with EGFR-mutant LUAD with high PD-L1 (≥50%) had shorter PFS (5.8 months) than non-EGFR/ALK LUAD patients who received chemoIO (12.8 months, *p* = 0.002; HR 0.22; 95% CI: 0.08–0.56) as first-line treatment. Chemotherapy-based therapy rendered similar benefit to patients with either EGFR exon19 deletion vs. L858R in the LUAD. Conclusions: This retrospective analysis revealed that immunotherapy provided limited additional benefit to chemotherapy in TKI-refractory EGFR-mutant LUAD. Chemotherapy alone or combined with bevacizumab remain good choices for patients with actionable EGFR mutations.

## 1. Introduction

Recent clinical studies have assessed the efficacy of combining immune checkpoint inhibitor therapy with standard first-line treatment of patients with metastatic non-small cell lung cancer (NSCLC). Specifically, chemoimmunotherapy has displayed a marked improvement in clinical outcome in these NSCLC patients. In the Keynote-189 trial, patients without sensitizing EGFR or ALK mutations were treated with pemetrexed and a platinum-based drug in combination with pembrolizumab (anti-PD1) or a placebo. This study found that the addition of anti-PD1 therapy improved both PFS from 4.9 months to 8.8 months and ORR from 18.9% to 47.7% [1]. Thus, platinum/pemetrexed/pembrolizumab is now widely used as the first-line therapy for patients with metastatic NSCLC without EGFR mutation or ALK fusions.

However, for NSCLC patients with EGFR mutations, it is not clear whether the addition of anti-PD1/L1 to chemotherapy as the initial treatment after TKI will render the same magnitude of benefit. As a monotherapy, anti-PD1/L1 therapy had limited benefit in EGFR-mutant NSCLC, with a response rate of less than 10% [2,3,4,5]. Even for patients whose EGFR-mutant lung cancer expressed high PD-L1 (>50%), the response was inferior to EGFR-wildtype high PD-L1 counterparts [6]. While the first-line treatment of choice for patients with EGFR-mutant NSCLC is an EGFR TKI, most patients eventually develop resistance to EGFR TKI therapy, making platinum-based chemotherapy the next line of therapy. Historically, platinum doublet chemotherapy has provided EGFR-mutant patients a PFS in the range of 5 months [1,7]. Most recently, one pooled analysis showed that the addition of anti-PD1/L1 immunotherapy to chemotherapy did not render significant additional benefit [8,9]. In contrast, Gadgeel et al. reported a median PFS of 8.3 months in EGFR-mutant NSCLC patients with platinum/pemetrexed/pembrolizumab [10], out-performing historical controls.

The vasculature architecture in the tumor microenvironment is a barrier constructed during tumor development that prevents anti-cancer attacks. Vascular endothelial growth factor (VEGF) is the major regulator in tumor angiogenesis. When combined with chemotherapy, anti-VEGF antibodies can alleviate hypoxia and acidosis, which contribute to chemotherapy resistance and improve outcomes of advanced NSCLC patients [11]. To date, two anti-VEGF monoclonal antibodies, bevacizumab and ramucirumab, have been globally approved for the treatment of NSCLC.

Furthermore, analysis of the Impower150 trial showed that EGFR-mutant NSCLC patients treated with a regimen of anti-PD-L1 (atezolizumab), anti-VEGF (bevacizumab), and carboplatin and paclitaxel had a PFS of over 10.2 months with response rate of 71% [12]. Final analysis from recently updated results of this trial continued to show an increase in OS benefit, notably in the patients treated with atezolizumab/bevacizumab/carboplatin/paclitaxel who had a sensitizing EGFR mutation with previous EGFR TKI failure [13]. Additionally, these patients had reduced risk of brain metastases development. Due to the limited sample size in this exploratory analysis, Nogami et al. and others have urged that the current findings be interpreted with caution pending findings from larger future clinical studies [13,14].

In our study, we sought to investigate whether the addition of anti-PD1/L1 to chemotherapy is beneficial for TKI-refractory EGFR-mutant NSCLC patients, what the potential contribution of the addition of anti-VEGF to chemotherapy is, and what the clinical features to predict clinical outcomes are. We performed a single-center retrospective analysis evaluating clinical outcomes of a cohort of 178 patients with EGFR-mutant lung adenocarcinoma (LUAD) post-EGFR TKI systemic therapy in patients who were treated at MD Anderson Cancer Center.

## 2. Materials and Methods

### 2.1. Study Population

The GEMINI-Moonshot Database collects information from lung cancer patients treated at the MD Anderson Cancer Center (MDACC), including demographics, cancer at diagnosis, treatment, molecular profiles, and outcomes. We queried GEMINI to identify patients with metastatic EGFR-mutant NSCLC who had progressed on EGFR TKIs and received subsequent non-TKI systemic therapy, from March 2014 to March 2021. All available clinical information was collected from electronic medical records. The data collection was performed under MDACC IRB-approved protocol PA13-0589. Review and validation were completed manually by investigators. Inclusion criteria for this retrospective cohort study include: (1) EGFR actionable mutation; (2) metastatic adenocarcinoma (LUAD); (3) progressed on EGFR-TKI; (4) more than one cycle of non-TKI systemic therapy after TKI treatment; (5) adequate clinical outcome data for analysis (Figure S1). Patients who maintained EGFR-TKI with post-TKI systemic treatment were not included in this study. This study was approved by the institutional review board at MDACC, and all patients provided written informed consent.

### 2.2. PD-L1 Expression and Genomic Profiling

The expression of PD-L1 was determined immunohistochemically using 22C3 pharmDx and quantified as tumor proportion score (TPS) at MDACC Molecular Diagnostics Laboratory. The molecular data were collected through pathology reports. Next-generation sequencing was used to determine the gene alterations of EGFR and TP53 in tumor tissue DNA (at MDACC Molecular Diagnostics Laboratory, Houston, TX, US or FoundationOne—Foundation Medicine Inc., Cambridge, MA, US) or circulating tumor DNA (HP MD Liquid Biopsy panel—70 or Guardant360 panel—Guardant Health Inc., Redwood City, CA, USA).

### 2.3. Statistical Analysis

The index year/date was defined as the starting year/date of subsequent non-TKI systemic treatment after progression on EGFR-TKI. Time-to-event outcomes used in this study were based on this date. Clinical progression-free survival (PFS) was calculated from the index date to disease progression by the physician’s judgment, or death, whichever occurred first. Overall survival (OS) was defined as the time from the index date to death from any cause. Patients alive or the absence of disease progression at last follow-up were censored for analyses. PFS and OS were estimated by the Kaplan–Meier method. Univariate and multicovariate Cox proportional hazards (PH) models were applied to further evaluate the association of covariates with survival outcomes. The PH assumption was checked using cox.zph function of the survival package in R. Survival rates at 6 and 12 months were expressed using descriptive statistics as proportions and compared by chi-squared test. All statistical analyses were performed on RStudio (version 2 September 2021), and *p* < 0.05 was considered statistically significant.

## 3. Results

### 3.1. Patient Population and Characteristics

We identified 732 NSCLC patients harboring EGFR mutation. Of these, 178 patients met the inclusion criteria and were eligible for this analysis (Figure S1). The clinical characteristics of study subjects were summarized in Table 1. All 178 patients were progressed on EGFR-TKI and treated with subsequent systemic non-TKI therapy. After TKI systemic therapy includes chemotherapy (chemo; *n* = 84), chemotherapy with immunotherapy (chemoIO; *n* = 30), chemotherapy plus bevacizumab with IO (chemoBevIO; *n* = 11) or without IO (chemoBev; *n* = 31), and IO monotherapy (IO-mono; *n* = 22) (Table 1). Immunotherapy was a new standard of care choice since 2014 and was incorporated with chemotherapy starting from 2017 (Figure S2). The median age at the time of systemic therapy after TKI was 63.3 (range: 27.4–83.3) years, 103 (57.9%) were female, and 102 (57.3%) were white patients. The median follow-up time was 42.0 (Interquartile range: 22.9–67.8) months. Total number of deaths from any cause during this period was 127 (71.3%).

### 3.2. Limited Benefit from the Addition of Immunotherapy to Chemotherapy

**Figure 1:**
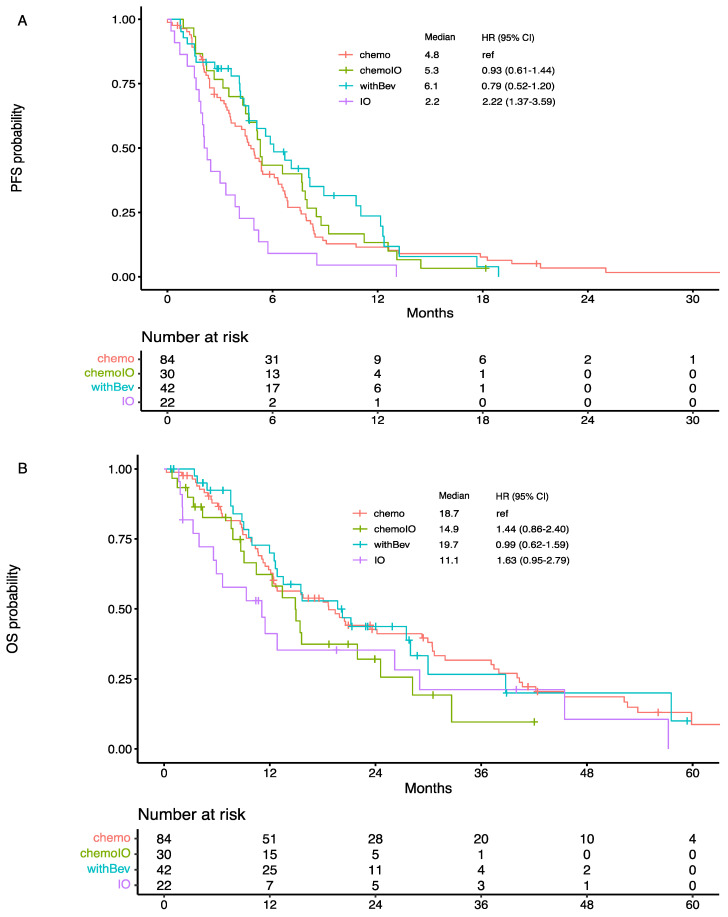
Clinical outcomes in TKI-refractory EGFR-mutant lung adenocarcinoma patients treated with subsequent regimen, including chemotherapy alone (chemo), chemotherapy combined with immunotherapy (chemoIO), chemotherapy plus bevacizumab with or without immunotherapy (withBev), and immunotherapy alone (IO). (**A**) Progression-free survival (PFS); (**B**) overall survival (OS).

A total of 63 patients were treated with immunotherapy alone or combined with chemotherapy, including 30 cases with chemoIO, 11 chemoBevIO, and 22 IO-mono. Most (76%) patients received anti-PD1-antibodies (pembrolizumab *n* = 39, nivolumab *n* = 9), while fewer patients (24%) received anti-PD-L1-antibodies (atezolizumab *n* = 12, durvalumab *n* = 3). The median PFS for chemoIO was 5.3 months (95% CI 4.47–7.97), compared to mPFS of 4.8 months (95% CI 3.63–6.30) with chemotherapy only, which showed no significant difference (*p* = 0.8, Figure 1A). When chemotherapy was used as the comparator, IO-mono was inferior (HR 2.22, *p* = 0.001), whereas chemoIO (HR 0.93, *p* = 0.8) or withBev (HR 0.79, *p* = 0.3) showed no statistical significance (Figure 1A). Overall survival (OS) showed no difference in all treatment groups, but the median OS was 14.9 (chemoIO) vs. 18.7 months (chemo, *p* = 0.2, Figure 1B), suggesting a trend toward chemoIO group being inferior to chemotherapy only. The clinical outcomes between chemoBev with IO and without IO showed no difference, acknowledging the small sample size (Figure S3).

The PFS rate at 6 months after starting post-TKI systemic therapy did not differ between chemo and chemoIO (39.8% [95% CI: 30.4–52.0%] vs. 43.3% [95% CI: 28.8–65.2%]; *p* = 0.6) nor at 12 months (11.6% [95% CI: 6.3–21.3%] vs. 13.3% [95% CI: 5.3–33.2%]; *p* = 0.4) (Figure 1A). Similarly, the difference of OS rate at 6 months was not statistically significant between chemo and chemoIO (87.8% [95% CI: 81.0–95.2%] vs. 82.7% [95% CI: 69.9–97.7%]; *p* = 0.4) nor at 12 months (64.0% [95% CI: 54.3–75.4%] vs. 62.3% [95% CI: 46.2–84.1%]; *p* = 0.7) (Figure 1B).

### 3.3. Inferior Response in High-PD-L1 EGFR-Mutant LUAD Compared to EGFR-Wildtype LUAD

**Figure 2:**
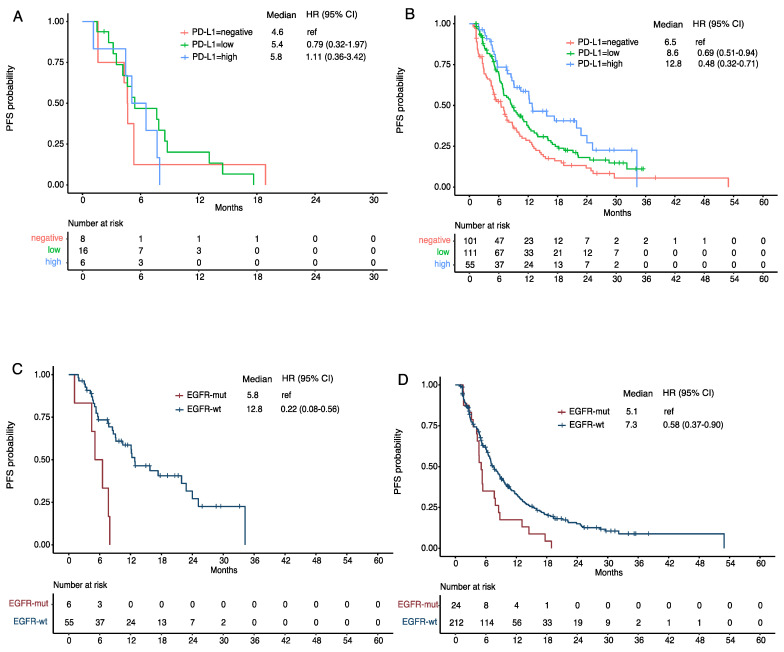
Progression-free survival (PFS) in lung adenocarcinoma patients with EGFR mutant (EGFR-mut) or EGFR wild type (EGFR-wt) who received chemotherapy-based immunotherapy as first-line. (**A**) PFS in EGFR-mutant patients stratified by PD-L1 level; (**B**) PFS in EGFR-wt patients stratified by PD-L1 level; (**C**) PFS in patients with high PD-L1 (TPS = 50–100%) between EGFR-mut and EGFR-wt; (**D**) PFS in patients with low or negative (TPS = 0–49%) PD-L1 level between EGFR-mut and EGFR-wt.

For the 30 patients who received chemoIO (with or without Bev) with available PD-L1 status, we stratified PFS by PD-L1 expression levels: <1% (negative, *n* = 8), 1–49% (low, *n* = 16), ≥50% (high, *n* = 6). The mPFS was 4.6, 5.4, and 5.8 months respectively (Figure 2A), with no difference among the groups (*p* = 0.76). In the same database of GEMINI, we also identified patients whose tumor did not have EGFR mutation or ALK fusion (non-EGFR/ALK) and received first-line chemoIO therapy for their metastatic LUAD (*n* = 267); mPFS was 6.5 months in PD-L1 negative group, 8.6 months in PD-L1 low group, and 12.8 months in the high group, respectively (Figure 2B). Although with small sample size, there was a striking PFS difference between EGFR-mutant vs. non-EGFR/ALK patients, especially in the high PD-L1 (≥ 50%) groups (5.8 vs. 12.8 months, *p* = 0.002, Figure 2C), indicating EGFR is a strong predictor of the lack of benefit in adding immunotherapy to chemotherapy, even in the high PD-L1 cases. In the negative-to-low PD-L1 groups, the difference was not as pronounced, although still significant (5.1 vs. 7.3 months, *p* = 0.014, Figure 2D). The prolongation of mPFS in the non-EGFR/ALK group could be related to the fact that EGFR-mutant lung cancer patients had prior targeted therapy and therefore, worse performance status and more tumor burden at start of chemoIO therapy. Similar trend was observed in OS between subgroups, although the differences were not significant (Figure S4).

### 3.4. Chemotherapy-Based Therapy Rendered Similar Benefit Regardless of EGFR or TP53 Mutation Patterns

It is known that EGFR Ex19Del responds to EGFR TKI better than L858R. In a prior analysis from GEMINI cohort, we have reported PFS with osimertinib to be 16.9 months in Ex19Del and 13.0 months in L858R, which was significantly different (HR 0.67 *p* = 0.005) [15]. In FLAURA trial with osimertinib used as the first-line treatment, the difference between two types of mutations was 7 months, with PFS of 21.4 months in Ex19Del and 14.4 in L858R group [16]. To understand whether there was a differential outcome with chemotherapy-based treatments in those two groups, we evaluated PFS and OS in Ex19Del (*n* = 95, 60.9%), L858R (*n* = 47, 30.1%), and other mutation (exon 20ins/G719X/T790M/L861Q) groups. No difference in PFS and OS was detected: the PFS for Ex19Del was 5.4 months (*n* = 95), L858R at 5.0 months (*n* = 47), and other mutations at 4.6 months (*n* = 14) (Figure S5A); the OS for Ex19Del was 19.7 months, L858R at 15.5 months, and other mutations at 18.9 months (*n* = 14) (Figure S5B).

Co-occurring mutation of TP53 was evaluated in the cohort with chemotherapy-based treatment (*n* = 156). Among this cohort, we found no significant difference in median PFS between TP53 mutation (*n* = 101) and TP53 wild type (*n* = 33), with 5.3 vs. 4.6 months (HR, 0.75; 95% CI, 0.49–1.15; *p* = 0.2, Figure S5C), and the median OS was 15.7 vs. 21.2 months (HR, 0.80; 95% CI, 0.50–1.26; *p* = 0.3; Figure S5D).

### 3.5. Liver Metastasis and Black Racial/Ethnic Group Predict the Worst Clinical Outcome

Last, we performed multicovariate analysis to identify high-risk patient groups. Covariates used for adjustment in the Cox regression model include age, gender, tobacco use, race, if only one type of TKI used, if the first line was osimertinib, the treatment strategy of the non-TKI systemic therapy, with or without liver metastases, with or without brain metastases, with or without bone metastases (Table 2). On multicovariate Cox regression (Table 2) for PFS among patients with chemotherapy-based treatment (*n* = 156), the following factors were significantly associated with an increased risk of progression: with liver metastasis (vs. without; 40 vs. 116; HR, 1.87; 95% CI: 1.17–2.99; *p* = 0.009), with brain metastasis (vs. without; 74 vs. 82; HR, 1.55; 95% CI: 1.05–2.3; *p* = 0.029). Liver metastasis at the time of chemotherapy-based treatment initiation was found in 22.6% (19) of 84 patients in the chemo group, 33.3% (10 out of 30 patients) in the chemoIO group, and 35.5% (11 out of 31 patients) in the chemoBev group (Table 1). Among the total cohort (*n* = 178), the first line of TKIs received included erlotinib (123 [69.1%]), osimertinib (30 [16.9%]), afatinib (13 [7.3%]), and gefitinib (12 [6.7%]). No statistical significance of clinical outcome on chemotherapy-based treatment was observed between patients with osimertinib and other generations as first line TKI. Receiving only one line of TKI therapy (oneTKIprior, *n* = 99) indicated a significantly decreased risk of progression on chemotherapy-based treatment vs. more than one prior TKI (*n* = 57; HR, 0.58; 95% CI: 0.37–0.92; *p* = 0.02). For OS among patients with chemotherapy-based treatment, the following factors were significantly associated with an increased all-cause mortality: age > 65 years old (vs. ≤ 65; 67 vs. 89; HR, 1.67; 95% CI: 1.1–2.55; *p* = 0.016), Black racial/ethnic group (vs. Asian group; 13 vs. 42; HR, 2.98; 95% CI: 1.34–6.62; *p* = 0.007), and with liver metastasis (vs. without; 40 vs. 116; HR, 1.9; 95% CI: 1.14–3.17; *p* = 0.014). No significant deviation of the PH assumption was noted.

## 4. Discussion

This retrospective study evaluated the efficacy of adding anti-PD1/L1 or anti-VEGF therapy to platinum-based chemotherapy in EGFR-TKI-resistant EGFR-mutant adenocarcinoma patients in a single center and real-world setting. We found that immunotherapy adds very limited benefit in terms of PFS and OS to platinum-based doublet. When stratified by PD-L1 levels, even patients with high PD-L1 expression (TPS 50–100%) did not derive as much benefit from chemoIO therapy in EGFR-mutant lung cancers, which is especially disappointing when compared to high PDL1 EGFR-wildtype tumors. This supports the possibility that EGFR mutation itself is a strong biomarker for not responding to chemoIO therapy.

The addition of anti-VEGF therapy showed a trend toward potential benefit compared with chemotherapy only (mPFS, 6.1 vs. 4.8 months, *p* = 0.3). A similar observation was also reported by White et al. in a retrospective analysis cohort [7]. VEGF expression can be upregulated in EGFR-mutant lung cancers, which is the underlying mechanism for enhancing the sensitivity of EGFR-mutant patients to bevacizumab or ramucirumab [17]. A number of clinical trials have demonstrated the benefit of anti-VEGF/EGFR double blockade, and ramucirumab–erlotinib combination has been approved by FDA for use for EGFR-mutant NSCLC. Along with chemotherapy, it is possible that anti-VEGF has a role to play in EGFR/VEGF signaling suppression.

The intriguing benefit of adding both anti-VEGF and anti-PD1/L1 to chemotherapy warrants further investigation. Anti-VEGF therapy can modulate the tumor immune microenvironment, as VEGF is an immune suppressive cytokine pathway. The combination of anti-VEGF and anti-PD1 therapy has shown promising efficacy both in the preclinical models and clinical trials [18]. When ramucirumab and pembrolizumab were combined, the ORR was 30% (95%CI 13.8–50.2) in a cohort of previously treated non-small cell lung cancer patients, preliminarily suggestive of benefit [19]. Furthermore, evidence from the use of VEGFR2 spectrum multi-targeting TKI sitravatinib in combination with nivolumab in the lung cancer patients who developed resistance to anti-PD1 therapy also indicates immune-modulation effect from VEGF targeting [20]. IMpower150 evaluated the efficacy of treatment combining IO, anti-VEGF, and chemotherapy in patients with progressed NSCLC [21]. An improvement in OS was distinguished in patients harboring EGFR mutations with the chemoBevIO group compared with the chemoBev (HR 0.31, 95% CI: 0.11–0.83). In the current cohort, the sample size of chemoBevIO is too small for comparison (*n* = 11).

In our cohort, liver metastasis is an independent risk factor for both PFS and OS with stronger HR than patients with CNS metastasis. Preclinical models have showed that the efficacy of immunotherapy was restrained in patients with liver metastasis by eliminating the amount of macrophage-mediated T cells [22]. Interestingly, in IMpower150 trial, the addition of atezolizumab and bevacizumab to the platinum–taxanes was beneficial to the patients with liver metastases [21]. However, immunotherapy alone or in combination with chemotherapy has shown minimal therapeutic benefit in this subgroup [23,24]. No clinical improvement was observed in the subgroup with liver metastases in IMpower130, indicating that the addition of anti-VEGF to the chemoIO may reverse the tissue-specific immunoregulation by EGFR mutation or liver metastases and is important in these patient subgroups. Based on the available data, the combined regimen might be the best option to be considered as the next-line treatment for patients with EGFR-mutation and liver metastases disease.

The mortality in patients with Black ethnicity is the highest among all racial/ethnic groups for most cancers [25]. No significant differences were found in clinically actionable genomic alterations between black and white populations with LUAD [26]. However, differences in access and quality of care likely contribute to the racial disparities. Although with limited sample size, black ethnicity (*n* = 11) is an independent risk factor of overall survival in the current cohort, although no significant difference was observed for PFS.

Our study was limited by the nature of a single-center retrospective cohort analysis; for example, PD-L1 results were only available in a limited number of patients. A larger multi-institutional collaboration is required to better understand the practice pattern and expand the sample size to further elucidate key questions raised here, including the benefit of anti-VEGF therapy, the optimal choice of therapy for patients with liver metastasis, and the mortality disparity in certain patient ethnic groups. Furthermore, although a Cox regression model was used to adjust for available characteristics, all possible confounding factors could not be controlled, such as comorbidities and changes in the standard care over years, which may impact outcomes. Ongoing trials are investigating immunotherapy with pemetrexed/platinum +/- bevacizumab on NSCLC patients with EGFR mutations after progression on EGFR-TKIs (the Keynote789 study [ClinicalTrials.gov number, NCT03515837], the CheckMate722 study [ClinicalTrials.gov number, NCT02864251], the TH-138 study [ClinicalTrials.gov number, NCT03786692]). These perspective studies will provide more data to make this point clearer.

## 5. Conclusions

We found that the addition of anti-PD1 immunotherapy did not add benefit to platinum-based chemotherapy at the time of TKI progression for EGFR-mutant LUAD. Although underpowered, the anti-VEGF therapy demonstrated a trend towards adding benefit. As clinical trials with newer agents or combinations demonstrate preliminary efficacy in TKI-resistant EGFR-mutant LUAD patients are ongoing, the ideal choice for post-TKI treatment is still being evaluated.

## Figures and Tables

**Table 1:** Characteristics of EGFR-mutant lung adenocarcinoma patients treated with non-TKI systemic therapy after TKI (*n* = 178).

Clinical Characteristic	Total (178)	Chemo (84)	ChemoIO (30)	ChemoBev (31)	ChemoBevIO (11)	IO-mono (22)
Age at chemo started						
≤65	101 (56.7)	43 (51.2)	14 (46.7)	23 (74.2)	9 (81.8)	12 (54.5)
>65	77 (43.3)	41 (48.8)	16 (53.3)	8 (25.8)	2 (18.2)	10 (45.5)
Gender						
Male	75 (42.1)	35 (41.7)	11 (36.7)	13 (41.9)	2 (18.2)	14 (63.6)
Female	103 (57.9)	49 (58.3)	19 (63.3)	18 (58.1)	9 (81.8)	8 (36.4)
Race/ethnicity						
Asian	42 (23.6)	22 (26.2)	10 (33.3)	7 (22.6)	3 (27.3)	0
Black	17 (9.6)	9 (10.7)	2 (6.7)	2 (6.5)	0	4 (18.2)
Hispanic/Latino	17 (9.6)	8 (9.5)	3 (10)	3 (9.7)	0	3 (13.6)
White	102 (57.3)	45 (53.6)	15(50)	19 (61.3)	8 (72.7)	15 (68.2)
Smoking						
Never	120 (67.4)	55 (65.5)	21 (70)	24 (77.4)	8 (72.7)	12 (54.5)
Formal/current	58 (32.6)	29 (34.5)	9 (30)	7 (22.6)	3 (27.3)	10 (45.5)
ECOG						
0–1	116 (65.2)	48 (57.1)	19 (63.3)	23 (74.2)	11	15 (68.2)
2–3	32 (18)	18 (21.4)	6 (20)	3 (9.7)	0	5 (22.7)
Unknown	30 (16.9)	18 (21.4)	5(16.7)	5 (16.1)	0	2 (9.1)
Metastasis site						
Brain	83 (46.6)	42 (50)	13 (43.3)	16 (51.6)	3 (27.3)	9(40.9)
Bone	109 (61.2)	50 (59.5)	19 (63.3)	19 (61.3)	7 (63.6)	14 (63.6)
Liver	47 (26.4)	19 (22.6)	10 (33.3)	11 (35.5)	0	7 (31.8)
No of prior TKI therapy						
1	108 (60.7)	60 (71.4)	9 (30)	23 (74.2)	7 (63.6)	9 (40.9)
>1	70 (39.3)	24 (28.6)	21 (70)	8 (25.8)	4 (36.4)	13 (59.1)
Osimertinib first line						
Yes	30 (16.9)	5 (6)	8 (26.7)	8 (25.8)	8 (72.7)	1 (4.5)
No	148 (83.1)	79 (94)	22 (73.3)	23 (74.2)	3 (27.3)	21 (95.5)
EGFR						
ex19DEL	108 (60.7)	50 (59.5)	17 (56.7)	21 (67.7)	7 (63.6)	13 (59.1)
L858R	52 (29.2)	25 (29.8)	11 (36.7)	7 (22.6)	4 (36.4)	5 (22.7)
others	18 (10.1)	9 (10.7)	2 (6.7)	3 (9.7)	0	4 (18.2)
TP53						
Wild type	39 (21.9)	20 (23.8)	5 (16.7)	6 (19.4)	2 (18.2)	6 (27.3)
Mutated	115 (64.6)	50 (59.5)	21 (70)	22 (71)	8 (72.7)	14 (63.6)
Unknown	24 (13.5)	14 (16.7)	4 (13.3)	3 (9.7)	1 (9.1)	2 (9.1)
PD-L1						
Negative	30 (16.9)	18 (21.4)	6 (20)	3 (9.7)	2 (18.2)	1 (4.5)
Low	37 (20.8)	10 (11.9)	11(36.7)	8 (25.8)	5 (45.5)	3 (13.6)
High	18 (10.1)	5 (6)	4 (13.3)	3 (9.7)	2 (18.2)	4 (18.2)
Unknown	93 (52.2)	51 (60.7)	9 (30)	17 (54.8)	2 (18.2)	14 (63.6)

Abbreviations: Chemo, chemotherapy; ChemoIO, chemotherapy with immunotherapy; ChemoBev, chemotherapy plus bevacizumab without immunotherapy; ChemoBevIO, chemotherapy plus bevacizumab with immunotherapy; IO-mono, IO monotherapy; ex19DEL, exon 19 deletion.

**Table 2:** Univariate and multicovariate Cox regression for progression-free survival (PFS) and overall survival (OS) among the chemotherapy-based treatment cohort (*n* = 156).

Parameters	PFS			OS		
	Unadjusted Hazard Ratio (95% CI)	Adjusted Hazard Ratio (95% CI)	Adjusted *p*-Value	Unadjusted Hazard Ratio (95% CI)	Adjusted Hazard Ratio (95% CI)	Adjusted *p*-Value
Age at chemo started						
≤65	1 (Reference)			1 (Reference)		
>65	1.08 (0.77–1.51)	1.13 (0.75–1.69)	0.558	1.64 (1.12–2.39)	1.67 (1.1–2.55)	0.016
Gender						
Female	1 (Reference)			1 (Reference)		
Male	0.90 (0.64–1.27)	1.03 (0.69–1.53)	0.898	0.94 (0.65–1.38)	1.13 (0.79–1.89)	0.358
Tobacco use						
Current/former	1 (Reference)			1 (Reference)		
Never	0.88 (0.61–1.26)	0.88 (0.59–1.31)	0.525	0.83 (0.56–1.25)	0.90 (0.58–1.39)	0.634
Race						
Asian	1 (Reference)			1 (Reference)		
White	1.09 (0.73–1.62)	0.85 (0.55–1.32)	0.463	1.35 (0.86–2.11)	1.20 (0.75–1.94)	0.442
Black	1.3 (0.67–2.52)	0.97 (0.47–2.00)	0.924	2.83 (1.36–5.88)	2.98 (1.34–6.62)	0.007
Hispanic/Latino	1.13 (0.61–2.51)	0.93 (0.48–1.78)	0.817	0.79 (0.38–1.62)	0.92 (0.43–1.98)	0.839
OneTKIprior *						
No	1 (Reference)			1 (Reference)		
Yes	0.73 (0.51–1.03)	0.58 (0.37–0.92)	0.020	0.70 (0.47–1.05)	0.67 (0.40–1.12)	0.128
Osimertinib first line						
No	1 (Reference)			1 (Reference)		
Yes	0.89 (0.56–1.41)	1.11 (0.62–1.96)	0.729	1.14 (0.66–1.95)	1.16 (0.59–2.26)	0.669
After_TKI						
Chemo only	1 (Reference)			1 (Reference)		
ChemoIO	0.93 (0.60–1.43)	0.67 (0.40–1.14)	0.141	1.46 (0.87–2.43)	1.10 (0.59–2.05)	0.754
ChemoBev	0.80 (0.50–1.29)	0.78 (0.46–1.31)	0.349	0.97 (0.58–1.63)	1.14 (0.65–1.99)	0.651
ChemoBevIO	0.76 (0.38–1.51)	0.85 (0.36–1.96)	0.696	1.13 (0.48–2.64)	1.57 (0.57–4.3)	0.381
Liver metastasis						
No	1 (Reference)			1 (Reference)		
Yes	1.87 (1.26–2.78)	1.87 (1.17–2.99)	0.009	2.06 (1.34–3.19)	1.9 (1.14–3.17)	0.014
Brain metastasis						
No	1 (Reference)			1 (Reference)		
Yes	1.61 (1.14–2.28)	1.55 (1.05–2.30)	0.029	1.32 (0.91–1.93)	1.28 (0.83–1.97)	0.264
Bone metastasis						
No	1 (Reference)			1 (Reference)		
Yes	1.28 (0.91–1.81)	1.09 (0.74–1.59)	0.667	1.90 (1.26–2.87)	1.47 (0.93–2.31)	0.099

* Whether patient was progressed on one type of EGFR-TKI before starting subsequent systemic non-TKI treatment.

## Data Availability

Relevant data supporting the findings of this study are available within the Article and Appendices, or are available from the authors upon reasonable request.

## Supplementary Materials

The following supporting information can be downloaded at: https://www.mdpi.com/article/10.3390/cancers14143473/s1, Figure S1: Consort diagram; Figure S2: Histogram of patients treated with after-TKI regimens by index year defined as the year of subsequent non-TKI systemic treatment started after progression on EGFR-TKI, colored by treatment strategies, including chemotherapy alone (chemo), chemotherapy combined with immunotherapy (chemoIO), chemotherapy plus bevacizumab (chemoBev), chemotherapy plus bevacizumab with immunotherapy (chemoBevIO), and immunotherapy alone (IO); Figure S3: Clinical outcomes in TKI-refractory EGFR-mutant lung adenocarcinoma patients treated with subsequent regimen, including chemotherapy alone (chemo), chemotherapy combined with immunotherapy (chemoIO), chemotherapy plus bevacizumab (chemoBev), chemotherapy plus bevacizumab with immunotherapy (chemoBevIO), and immunotherapy alone (IO). (A) progression-free survival (PFS); (B) overall survival (OS); Figure S4: Overall survival (OS) in lung adenocarcinoma patients with EGFR mutant (EGFR-mut) or EGFR wild type (EGFR-wt) received chemotherapy-based immunotherapy as first line. (A) OS in EGFR-mut patients stratified by PD-L1 level; (B) OS in EGFR-wt patients stratified by PD-L1 level; (C) OS in patients with high PD-L1 (TPS = 50%-100%) between EGFR-mut and EGFR-wt; (D) OS in patients with low or negative (TPS = 0%-49%) PD-L1 level between EGFR-mut and EGFR-wt; Figure S5: Association between EGFR or TP53 alterations and clinical outcomes in TKI-refractory EGFR-mutant lung adenocarcinoma patients treated with chemotherapy-based regimen. (A) Progression-free survival (PFS) stratified by EGFR alterations. (B) Overall survival (OS) stratified by EGFR alterations. (C) PFS in patients with different TP53 status: mutation, unknown, or wild type (WT). (D) OS in patients with different TP53 status: mutation, unknown, or wild type (WT).

## References

[1] Gandhi L., Rodriguez-Abreu D., Gadgeel S., Esteban E., Felip E., De Angelis F., Domine M., Clingan P., Hochmair M.J., Powell S.F. (2018). Pembrolizumab plus Chemotherapy in Metastatic Non-Small-Cell Lung Cancer. N. Engl. J. Med..

[2] Lee C.K., Man J., Lord S., Links M., Gebski V., Mok T., Yang J.C. (2017). Checkpoint Inhibitors in Metastatic EGFR-Mutated Non-Small Cell Lung Cancer-A Meta-Analysis. J. Thorac. Oncol..

[3] Herbst R.S., Baas P., Kim D.W., Felip E., Perez-Gracia J.L., Han J.Y., Molina J., Kim J.H., Arvis C.D., Ahn M.J. (2016). Pembrolizumab versus docetaxel for previously treated, PD-L1-positive, advanced non-small-cell lung cancer (KEYNOTE-010): A randomised controlled trial. Lancet.

[4] Rittmeyer A., Barlesi F., Waterkamp D., Park K., Ciardiello F., von Pawel J., Gadgeel S.M., Hida T., Kowalski D.M., Dols M.C. (2017). Atezolizumab versus docetaxel in patients with previously treated non-small-cell lung cancer (OAK): A phase 3, open-label, multicentre randomised controlled trial. Lancet.

[5] Borghaei H., Paz-Ares L., Horn L., Spigel D.R., Steins M., Ready N.E., Chow L.Q., Vokes E.E., Felip E., Holgado E. (2015). Nivolumab versus Docetaxel in Advanced Nonsquamous Non-Small-Cell Lung Cancer. N. Engl. J. Med..

[6] Gainor J.F., Shaw A.T., Sequist L.V., Fu X., Azzoli C.G., Piotrowska Z., Huynh T.G., Zhao L., Fulton L., Schultz K.R. (2016). EGFR Mutations and ALK Rearrangements Are Associated with Low Response Rates to PD-1 Pathway Blockade in Non-Small Cell Lung Cancer: A Retrospective Analysis. Clin. Cancer Res..

[7] White M.N., Piper-Vallillo A.J., Gardner R.M., Cunanan K., Neal J.W., Das M., Padda S.K., Ramchandran K., Chen T.T., Sequist L.V. (2022). Chemotherapy Plus Immunotherapy Versus Chemotherapy Plus Bevacizumab Versus Chemotherapy alone in EGFR-Mutant NSCLC after Progression on Osimertinib. Clin. Lung Cancer.

[8] Aredo J.V., Mambetsariev I., Hellyer J.A., Amini A., Neal J.W., Padda S.K., McCoach C.E., Riess J.W., Cabebe E.C., Naidoo J. (2021). Durvalumab for Stage III EGFR-Mutated NSCLC after Definitive Chemoradiotherapy. J. Thorac. Oncol..

[9] Aredo J.V., Hellyer J.A., Neal J.W., Wakelee H.A. (2021). Consolidation Durvalumab Should Not Be Administered to Patients with Stage III EGFR-Mutant NSCLC. J. Thorac. Oncol..

[10] Gadgeel S., Dziubek K., Nagasaka M., Braun T., Hassan K., Cheng H., Wozniak A., Halmos B., Stevenson J., Patil P. (2021). Pembrolizumab in Combination with Platinum-Based Chemotherapy in Recurrent EGFR/ALK-Positive Non-Small Cell Lung Cancer (NSCLC). J. Thorac. Oncol..

[11] Morbidelli L. (2022). Antiangiogenic Drugs as Chemosensitizers in Cancer Therapy.

[12] Socinski M.A., Jotte R.M., Cappuzzo F., Orlandi F., Stroyakovskiy D., Nogami N., Rodriguez-Abreu D., Moro-Sibilot D., Thomas C.A., Barlesi F. (2018). Atezolizumab for First-Line Treatment of Metastatic Nonsquamous NSCLC. N. Engl. J. Med..

[13] Nogami N., Barlesi F., Socinski M.A., Reck M., Thomas C.A., Cappuzzo F., Mok T.S.K., Finley G., Aerts J.G., Orlandi F. (2022). IMpower150 Final Exploratory Analyses for Atezolizumab Plus Bevacizumab and Chemotherapy in Key NSCLC Patient Subgroups with EGFR Mutations or Metastases in the Liver or Brain. J. Thorac. Oncol..

[14] Muthusamy B., Pennell N. (2022). Chemoimmunotherapy for EGFR-Mutant NSCLC: Still No Clear Answer. J. Thorac. Oncol..

[15] Liu X., Hong L., Nilsson M., Hubert S.M., Wu S., Rinsurongkawong W., Lewis J., Spelman A., Roth J., Swisher S. (2020). Concurrent use of aspirin with osimertinib is associated with improved survival in advanced EGFR-mutant non-small cell lung cancer. Lung Cancer.

[16] Soria J.C., Ohe Y., Vansteenkiste J., Reungwetwattana T., Chewaskulyong B., Lee K.H., Dechaphunkul A., Imamura F., Nogami N., Kurata T. (2018). Osimertinib in Untreated EGFR-Mutated Advanced Non-Small-Cell Lung Cancer. N. Engl. J. Med..

[17] Hung M.S., Chen I.C., Lin P.Y., Lung J.H., Li Y.C., Lin Y.C., Yang C.T., Tsai Y.H. (2016). Epidermal growth factor receptor mutation enhances expression of vascular endothelial growth factor in lung cancer. Oncol. Lett..

[18] Manegold C., Dingemans A.C., Gray J.E., Nakagawa K., Nicolson M., Peters S., Reck M., Wu Y.L., Brustugun O.T., Crino L. (2017). The Potential of Combined Immunotherapy and Antiangiogenesis for the Synergistic Treatment of Advanced NSCLC. J. Thorac. Oncol..

[19] Herbst R.S., Arkenau H.T., Santana-Davila R., Calvo E., Paz-Ares L., Cassier P.A., Bendell J., Penel N., Krebs M.G., Martin-Liberal J. (2019). Ramucirumab plus pembrolizumab in patients with previously treated advanced non-small-cell lung cancer, gastro-oesophageal cancer, or urothelial carcinomas (JVDF): A multicohort, non-randomised, open-label, phase 1a/b trial. Lancet Oncol.

[20] Leal T.A., Berz D., Rybkin I., Iams W.T., Bruno D., Blakely C., Spira A., Patel M.R., Waterhouse D.M., Richards D. (2021). MRTX-500: Phase II trial of sitravatinib (sitra) + nivolumab (nivo) in patients (pts) with non-squamous (NSQ) non-small cell lung cancer (NSCLC) progressing on or after prior checkpoint inhibitor (CPI) therapy. ESMO Congr. Ann. Oncol..

[21] Reck M., Mok T.S.K., Nishio M., Jotte R.M., Cappuzzo F., Orlandi F., Stroyakovskiy D., Nogami N., Rodriguez-Abreu D., Moro-Sibilot D. (2019). Atezolizumab plus bevacizumab and chemotherapy in non-small-cell lung cancer (IMpower150): Key subgroup analyses of patients with EGFR mutations or baseline liver metastases in a randomised, open-label phase 3 trial. Lancet Respir. Med..

[22] Yu J., Green M.D., Li S., Sun Y., Journey S.N., Choi J.E., Rizvi S.M., Qin A., Waninger J.J., Lang X. (2021). Liver metastasis restrains immunotherapy efficacy via macrophage-mediated T cell elimination. Nat. Med..

[23] Tumeh P.C., Hellmann M.D., Hamid O., Tsai K.K., Loo K.L., Gubens M.A., Rosenblum M., Harview C.L., Taube J.M., Handley N. (2017). Liver Metastasis and Treatment Outcome with Anti-PD-1 Monoclonal Antibody in Patients with Melanoma and NSCLC. Cancer Immunol. Res..

[24] West H., McCleod M., Hussein M., Morabito A., Rittmeyer A., Conter H.J., Kopp H.G., Daniel D., McCune S., Mekhail T. (2019). Atezolizumab in combination with carboplatin plus nab-paclitaxel chemotherapy compared with chemotherapy alone as first-line treatment for metastatic non-squamous non-small-cell lung cancer (IMpower130): A multicentre, randomised, open-label, phase 3 trial. Lancet Oncol..

[25] Giaquinto A.N., Miller K.D., Tossas K.Y., Winn R.A., Jemal A., Siegel R.L. (2022). Cancer statistics for African American/Black People 2022. CA Cancer J. Clin..

[26] Campbell J.D., Lathan C., Sholl L., Ducar M., Vega M., Sunkavalli A., Lin L., Hanna M., Schubert L., Thorner A. (2017). Comparison of Prevalence and Types of Mutations in Lung Cancers Among Black and White Populations. JAMA Oncol..

