# Supplementary material for: Limited Benefit from the Addition of Immunotherapy to Chemotherapy in TKI-Refractory EGFR-Mutant Lung Adenocarcinoma
Source: Cancers (Basel). 2022 Jul 17;14(14):3473. doi: 10.3390/cancers14143473 (PMC9323840; doi:10.3390/cancers14143473)
Supplement: Supplementary file 1 [file cancers-14-03473-s001.zip › cancers-1786285-supplementary.pdf]

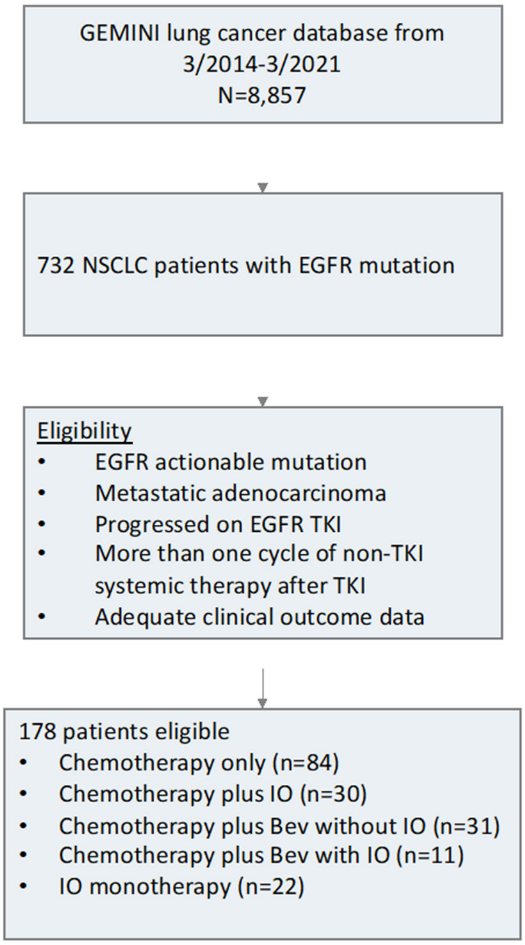

Figure S1. Consort diagram.

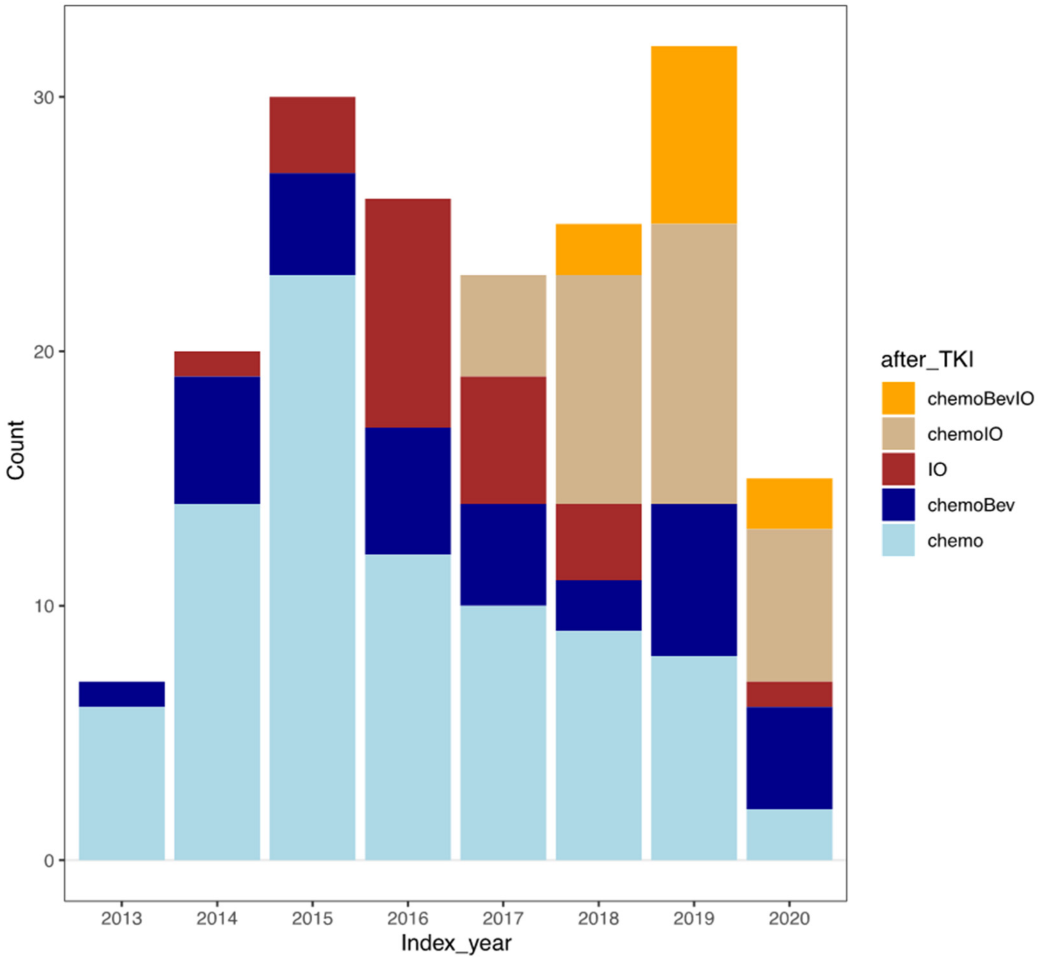

Figure S2. Histogram of patients treated with after-TKI regimens by index year, defined as the year of subsequent non-TKI systemic treatment started after progression on EGFR-TKI. Coloring depcits treatment strategies, including chemotherapy alone (chemo), chemotherapy combined with immunotherapy (chemoIO), chemotherapy plus bevacizumab (chemoBev), chemotherapy plus bevacizumab with immunotherapy (chemoBevIO), and immunotherapy alone (IO).

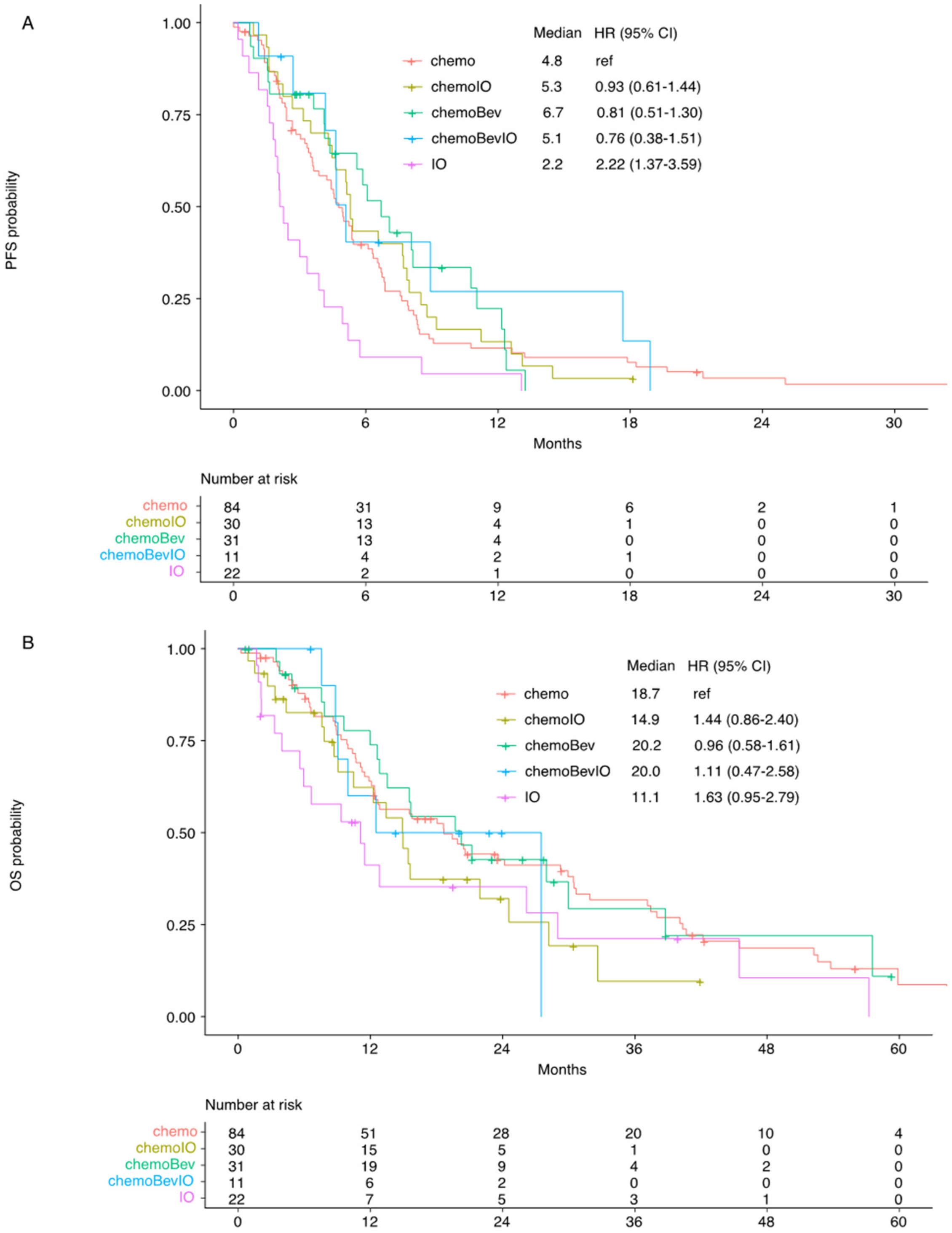

Figure S3. Clinical outcomes in TKI-refractory EGFR-mutant lung adenocarcinoma patients treated with subsequent regimen, including chemotherapy alone (chemo), chemotherapy combined with immunotherapy (chemoIO), chemotherapy plus bevacizumab (chemoBev), chemotherapy plus bevacizumab with immunotherapy (chemoBevIO), and immunotherapy alone (IO). (A) Progression-free survival (PFS); (B) overall survival (OS).

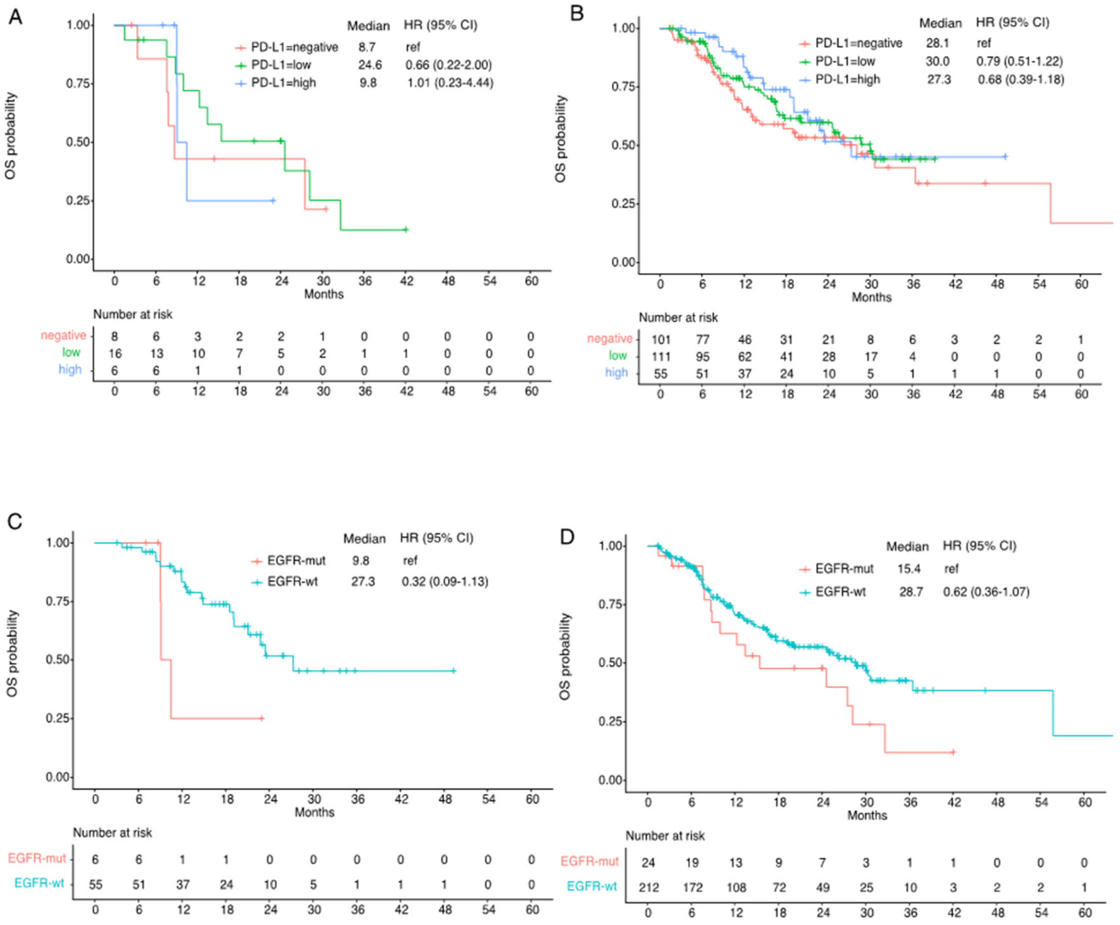

Figure S4. Overall survival (OS) in lung adenocarcinoma patients with EGFR mutant (EGFR-mut) or EGFR wildtype (EGFR-wt) received chemotherapy-based immunotherapy as first-line. (A) OS in EGFR-mut patients stratified by PD-L1 level; (B) OS in EGFR-wt patients stratified by PD-L1 level; (C) OS in patients with high PD-L1 (TPS = 50%-100%) between EGFR-mut and EGFR-wt; (D) OS in patients with low or negative (TPS = 0%-49%) PD-L1 level between EGFR-mut and EGFR-wt.

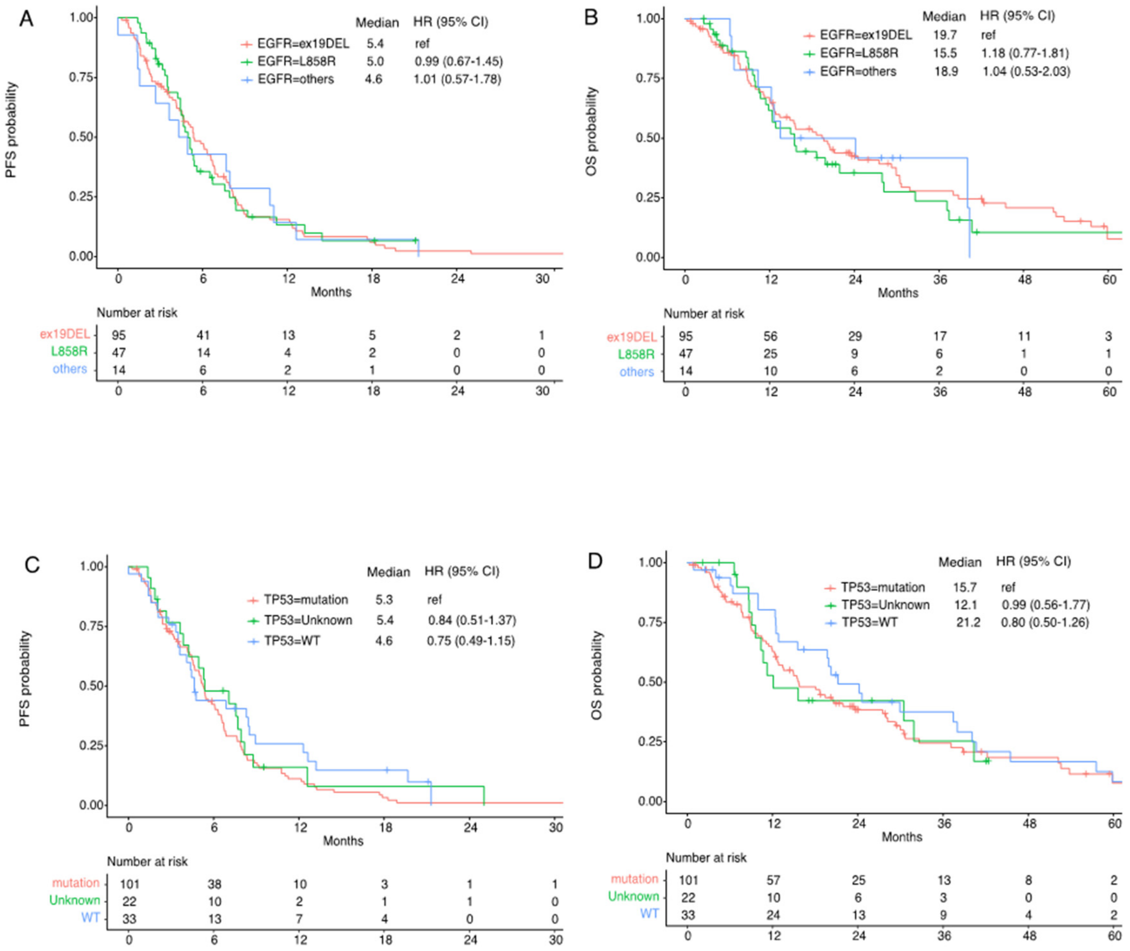

Figure S5. Association between EGFR or TP53 alterations and clinical outcomes in TKI-refractory EGFR-mutant lung adenocarcinoma patients treated with chemotherapy-based regimen. (A) Progression-free survival (PFS) stratified by EGFR alterations. (B) Overall survival (OS) stratified by EGFR alterations. (C) PFS in patients with different TP53 status: mutation, unknown, or wildtype (WT). (D) OS in patients with different TP53 status: mutation, unknown, or wildtype (WT).
